# Ethyl 1-*tert*-butyl-2-(4-methoxy­phen­yl)-1*H*-benzimidazole-5-carboxyl­ate

**DOI:** 10.1107/S1600536810007956

**Published:** 2010-03-06

**Authors:** Natarajan Arumugam, Shafida Abd Hamid, Aisyah Saad Abdul Rahim, Madhukar Hemamalini, Hoong-Kun Fun

**Affiliations:** a School of Pharmaceutical Sciences, Universiti Sains Malaysia, 11800 USM, Penang, Malaysia; b Kulliyyah of Science, International Islamic University Malaysia (IIUM), Jalan Istana, Bandar Indera Mahkota, 25200 Kuantan, Pahang, Malaysia; c X-ray Crystallography Unit, School of Physics, Universiti Sains Malaysia, 11800 USM, Penang, Malaysia

## Abstract

In the title mol­ecule, C_21_H_24_N_2_O_3_, the imidazole ring is essentially planar, with a maxium deviation of 0.015 (1) Å. The dihedral angle between the benzene and imidazole rings is 65.47 (6)°. The crystal packing is stabilized by weak inter­molecular C—H⋯O and C—H⋯N hydrogen bonds, forming zigzag chains along the *c* axis. The crystal structure is further stabilized by C—H⋯π inter­actions.

## Related literature

For background to benzimidazole derivatives, their biological activity and medical applications, see: Orjales *et al.* (1997 ▶); Andrzejewska *et al.* (2002 ▶); Garuti *et al.* (2000 ▶); Lukevics *et al.* (2001 ▶); Komazin *et al.* (2003 ▶). For details of hydrogen bonding, see: Jeffrey & Saenger (1991 ▶); Jeffrey (1997 ▶); Scheiner (1997 ▶). For the stability of the temperature controller used in the data collection, see: Cosier & Glazer (1986 ▶). 
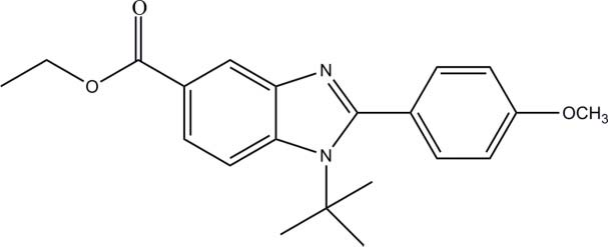


## Experimental

### 

#### Crystal data

C_21_H_24_N_2_O_3_
*M* *_r_* = 352.42
Orthorhombic, 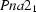

*a* = 14.3963 (7) Å
*b* = 8.6206 (5) Å
*c* = 15.1609 (8) Å
*V* = 1881.54 (17) Å^3^
*Z* = 4
Mo *K*α radiation
μ = 0.08 mm^−1^
*T* = 100 K
0.53 × 0.42 × 0.27 mm

#### Data collection

Bruker APEX DUO CCD area-detector diffractometer
Absorption correction: multi-scan (*SADABS*; Bruker, 2009 ▶) *T*_min_ = 0.957, *T*_max_ = 0.978
17426 measured reflections
4233 independent reflections
3951 reflections with *I* > 2σ(*I*)
*R*_int_ = 0.032

#### Refinement

*R*[*F* ^2^ > 2σ(*F* ^2^)] = 0.037
*wR*(*F* ^2^) = 0.113
*S* = 1.13
4233 reflections
240 parameters
1 restraint
H-atom parameters constrained
Δρ_max_ = 0.60 e Å^−3^
Δρ_min_ = −0.57 e Å^−3^

### 

Data collection: *APEX2* (Bruker, 2009 ▶); cell refinement: *SAINT* (Bruker, 2009 ▶); data reduction: *SAINT*; program(s) used to solve structure: *SHELXTL* (Sheldrick, 2008 ▶); program(s) used to refine structure: *SHELXTL*; molecular graphics: *SHELXTL*; software used to prepare material for publication: *SHELXTL* and *PLATON* (Spek, 2009 ▶).

## Supplementary Material

Crystal structure: contains datablocks global, I. DOI: 10.1107/S1600536810007956/wn2378sup1.cif

Structure factors: contains datablocks I. DOI: 10.1107/S1600536810007956/wn2378Isup2.hkl

Additional supplementary materials:  crystallographic information; 3D view; checkCIF report

## Figures and Tables

**Table 1:** Hydrogen-bond geometry (Å, °) *Cg*1, *Cg*2 and *Cg*3 are the centroids of the rings N1,N2,C7–C9 and C1–C6 and C8–C13, respectively.

*D*—H⋯*A*	*D*—H	H⋯*A*	*D*⋯*A*	*D*—H⋯*A*
C5—H5*A*⋯O2^i^	0.93	2.57	3.3822 (16)	146
C13—H13*A*⋯O2^ii^	0.93	2.52	3.4116 (16)	160
C19—H19*C*⋯N2^iii^	0.96	2.57	3.4976 (19)	164
C2—H2*A*⋯*Cg*3^iv^	0.93	2.75	3.5594 (14)	146
C18—H18*C*⋯*Cg*2^v^	0.96	2.74	3.6878 (16)	168
C21—H21*B*⋯*Cg*1^iv^	0.96	2.78	3.4703 (16)	130

Symmetry codes: (i) 
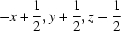
; (ii) 
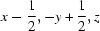
; (iii) 
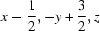
; (iv) 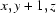; (v) 
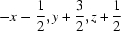
.

**Fig. 1.**
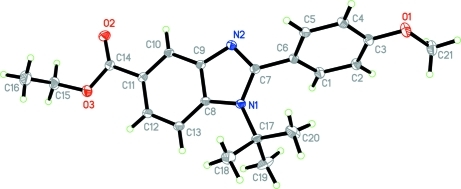
The asymmetric unit of the title compound, showing 50% probability displacement ellipsoids and the atom-numbering scheme. Hydrogen atoms are shown as spheres of arbitrary radius.

**Fig. 2.**
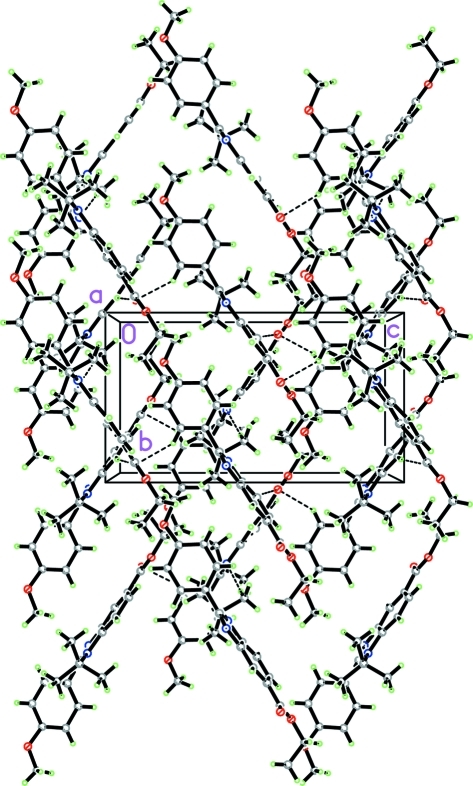
The crystal packing of the title compound, showing the hydrogen-bonded (dashed lines) network. H atoms not involved in the hydrogen bond interactions have been omitted for clarity.

## supplementary crystallographic information

### Comment

Benzimidazole derivatives are reported to be physiologically and pharmacologically active and find applications in the treatment of several diseases, such as epilepsy, diabetes and infertility (Orjales *et al.*, 1997). In addition, they also show clinical benefit toward breast cancer (Andrzejewska *et al.*, 2002), leukemia (Garuti *et al.*, 2000), tumor cells (Lukevics *et al.*, 2001) and possess potent antiviral activities (Komazin *et al.*, 2003). We present here the crystal structure of the title compound.

In the asymmetric unit of the title compound (Fig. 1), the imidazole ring is essentially planar, with a maximum deviation of 0.015 (1) Å for atom C8. The dihedral angle between the imidazole ring (N1/N2/C7–C9) and the benzene ring (C1–C6) is 65.47 (6)° .

In the crystal structure (Fig. 2), neighbouring molecules are connected by weak intermolecular C5—H5A···O2, C13—H13A···O2 and C19—H19C···N2  hydrogen bonds (Jeffrey & Saenger, 1991; Jeffrey, 1997; Scheiner, 1997), forming zigzag chains along the c-axis. The crystal structure is further stabilized by C—H···π interactions (Table 1), involving the N1/N2/C7–C9 (centroid Cg1), C1–C6 (centroid Cg2) and C8–C13 (centroid Cg3) rings.

### Experimental

Ethyl-3-amino-4-(*tert*-butylamino) benzoate (200 mg, 0.84 mmol) and the sodium metabisulfite adduct of 4-methoxybenzaldehyde (406 mg, 1.68 mmol) were dissolved in DMF. The reaction mixture was irradiated under microwave conditions at 130 °C for 2 minutes. After completion, the reaction mixture was diluted in EtOAc (20 ml) and washed with H_2_O (20 ml).  The organic layer was collected, dried over Na_2_SO_4_ and then evaporated in vacuo to yield the crude product. The product was recrystallised from EtOAc as colourless crystals.

### Refinement

All hydrogen atoms were positioned geometrically [C—H = 0.93 - 0.97 Å] and were refined using a riding model, with *U*_iso_(H) = x*U*_eq_(C), where x = 1.5 for methyl H and 1.2 for all other H atoms. A rotating group model was applied to the methyl groups. In the absence of significant anomalous scattering effects, 3066 Friedel pairs were merged.

### Crystal data

**Table:**

C_21_H_24_N_2_O_3_	*F*(000) = 752
*M**_r_* = 352.42	*D*_x_ = 1.244 Mg m^−^^3^
Orthorhombic, *P**n**a*2_1_	Mo *K*α radiation, λ = 0.71073 Å
Hall symbol: P 2c -2n	Cell parameters from 6765 reflections
*a* = 14.3963 (7) Å	θ = 2.7–36.9°
*b* = 8.6206 (5) Å	µ = 0.08 mm^−^^1^
*c* = 15.1609 (8) Å	*T* = 100 K
*V* = 1881.54 (17) Å^3^	Block, colourless
*Z* = 4	0.53 × 0.42 × 0.27 mm

### Data collection

**Table:**

Bruker APEX DUO CCD area-detector diffractometer	4233 independent reflections
Radiation source: fine-focus sealed tube	3951 reflections with *I* > 2σ(*I*)
graphite	*R*_int_ = 0.032
φ and ω scans	θ_max_ = 35.0°, θ_min_ = 2.7°
Absorption correction: multi-scan (*SADABS*; Bruker, 2009)	*h* = −23→12
*T*_min_ = 0.957, *T*_max_ = 0.978	*k* = −12→13
17426 measured reflections	*l* = −24→21

### Refinement

**Table:**

Refinement on *F*^2^	Primary atom site location: structure-invariant direct methods
Least-squares matrix: full	Secondary atom site location: difference Fourier map
*R*[*F*^2^ > 2σ(*F*^2^)] = 0.037	Hydrogen site location: inferred from neighbouring sites
*wR*(*F*^2^) = 0.113	H-atom parameters constrained
*S* = 1.13	*w* = 1/[σ^2^(*F*_o_^2^) + (0.0773*P*)^2^] where *P* = (*F*_o_^2^ + 2*F*_c_^2^)/3
4233 reflections	(Δ/σ)_max_ = 0.001
240 parameters	Δρ_max_ = 0.60 e Å^−^^3^
1 restraint	Δρ_min_ = −0.57 e Å^−^^3^

### Special details

**Table:**

Experimental. The crystal was placed in the cold stream of an Oxford Cryosystems Cobra open-flow nitrogen cryostat (Cosier & Glazer, 1986) operating at 100.0 (1) K.
Geometry. All s.u.'s (except the s.u. in the dihedral angle between two l.s. planes) are estimated using the full covariance matrix. The cell s.u.'s are taken into account individually in the estimation of s.u.'s in distances, angles and torsion angles; correlations between s.u.'s in cell parameters are only used when they are defined by crystal symmetry. An approximate (isotropic) treatment of cell s.u.'s is used for estimating s.u.'s involving l.s. planes.
Refinement. Refinement of F^2^ against ALL reflections. The weighted R-factor wR and goodness of fit S are based on F^2^, conventional R-factors R are based on F, with F set to zero for negative F^2^. The threshold expression of F^2^ > 2σ(F^2^) is used only for calculating R-factors(gt) etc. and is not relevant to the choice of reflections for refinement. R-factors based on F^2^ are statistically about twice as large as those based on F, and R- factors based on ALL data will be even larger.

### Fractional atomic coordinates and isotropic or equivalent isotropic displacement parameters (Å^2^)

**Table:**

	*x*	*y*	*z*	*U*_iso_*/*U*_eq_	
O1	0.12374 (8)	1.21773 (11)	0.18521 (7)	0.02061 (19)	
O2	0.25619 (7)	0.09272 (11)	0.59077 (8)	0.02019 (19)	
O3	0.11256 (7)	0.01389 (12)	0.63146 (8)	0.02132 (19)	
N1	0.00387 (7)	0.60189 (11)	0.40798 (7)	0.01307 (17)	
N2	0.16125 (7)	0.58244 (12)	0.40296 (7)	0.01439 (18)	
C1	0.07671 (9)	0.95165 (14)	0.37017 (8)	0.0159 (2)	
H1A	0.0560	0.9522	0.4283	0.019*	
C2	0.08488 (9)	1.09163 (14)	0.32461 (9)	0.0158 (2)	
H2A	0.0694	1.1849	0.3518	0.019*	
C3	0.11669 (9)	1.08946 (13)	0.23757 (8)	0.01465 (19)	
C4	0.14432 (9)	0.95031 (14)	0.19836 (8)	0.0156 (2)	
H4A	0.1685	0.9503	0.1415	0.019*	
C5	0.13556 (8)	0.81218 (14)	0.24449 (8)	0.01461 (19)	
H5A	0.1541	0.7195	0.2184	0.018*	
C6	0.09897 (8)	0.81128 (13)	0.33013 (8)	0.01312 (18)	
C7	0.08802 (8)	0.66340 (14)	0.37851 (8)	0.01293 (19)	
C8	0.02836 (8)	0.47467 (13)	0.45937 (8)	0.01276 (18)	
C9	0.12598 (8)	0.46271 (13)	0.45378 (8)	0.01270 (18)	
C10	0.17417 (8)	0.34437 (14)	0.49659 (8)	0.01382 (19)	
H10A	0.2384	0.3360	0.4921	0.017*	
C11	0.12284 (8)	0.23910 (13)	0.54632 (8)	0.01365 (18)	
C12	0.02572 (8)	0.25487 (14)	0.55447 (8)	0.0156 (2)	
H12A	−0.0068	0.1845	0.5892	0.019*	
C13	−0.02274 (8)	0.37223 (14)	0.51221 (8)	0.0157 (2)	
H13A	−0.0867	0.3826	0.5187	0.019*	
C14	0.17224 (8)	0.10981 (14)	0.59068 (9)	0.01472 (19)	
C15	0.14999 (11)	−0.12009 (16)	0.67719 (10)	0.0227 (2)	
H15A	0.1157	−0.1375	0.7314	0.027*	
H15B	0.2145	−0.1017	0.6923	0.027*	
C16	0.14263 (14)	−0.26002 (18)	0.61893 (12)	0.0300 (3)	
H16A	0.1631	−0.3501	0.6507	0.045*	
H16B	0.1809	−0.2458	0.5677	0.045*	
H16C	0.0792	−0.2738	0.6010	0.045*	
C17	−0.09468 (8)	0.64670 (15)	0.38647 (9)	0.0157 (2)	
C18	−0.14919 (10)	0.50058 (19)	0.36114 (13)	0.0279 (3)	
H18A	−0.1544	0.4337	0.4115	0.042*	
H18B	−0.1172	0.4471	0.3146	0.042*	
H18C	−0.2101	0.5294	0.3414	0.042*	
C19	−0.13666 (11)	0.7233 (2)	0.46773 (11)	0.0290 (3)	
H19A	−0.0993	0.8106	0.4846	0.044*	
H19B	−0.1386	0.6499	0.5153	0.044*	
H19C	−0.1985	0.7578	0.4545	0.044*	
C20	−0.10132 (11)	0.7557 (2)	0.30761 (12)	0.0305 (4)	
H20A	−0.0752	0.8545	0.3229	0.046*	
H20B	−0.1653	0.7688	0.2914	0.046*	
H20C	−0.0677	0.7124	0.2588	0.046*	
C21	0.09176 (12)	1.36157 (16)	0.22065 (10)	0.0232 (3)	
H21A	0.0965	1.4409	0.1765	0.035*	
H21B	0.1291	1.3894	0.2707	0.035*	
H21C	0.0281	1.3511	0.2387	0.035*	

### Atomic displacement parameters (Å^2^)

**Table:**

	*U*^11^	*U*^22^	*U*^33^	*U*^12^	*U*^13^	*U*^23^
O1	0.0309 (5)	0.0144 (4)	0.0165 (4)	−0.0019 (3)	0.0026 (4)	0.0018 (3)
O2	0.0146 (4)	0.0221 (4)	0.0239 (4)	0.0031 (3)	−0.0012 (3)	0.0034 (4)
O3	0.0189 (4)	0.0182 (4)	0.0269 (5)	0.0009 (3)	0.0004 (4)	0.0079 (4)
N1	0.0101 (4)	0.0151 (4)	0.0141 (4)	0.0004 (3)	0.0000 (3)	0.0013 (3)
N2	0.0117 (4)	0.0141 (4)	0.0174 (4)	−0.0014 (3)	0.0000 (3)	0.0012 (3)
C1	0.0196 (5)	0.0144 (5)	0.0138 (5)	−0.0004 (4)	0.0016 (4)	−0.0007 (4)
C2	0.0195 (5)	0.0135 (5)	0.0145 (5)	−0.0007 (4)	0.0013 (4)	−0.0010 (4)
C3	0.0165 (5)	0.0137 (4)	0.0137 (5)	−0.0022 (4)	−0.0007 (4)	−0.0003 (4)
C4	0.0172 (5)	0.0162 (5)	0.0134 (5)	−0.0025 (4)	0.0015 (4)	−0.0012 (4)
C5	0.0140 (4)	0.0142 (4)	0.0156 (5)	−0.0008 (4)	0.0012 (4)	−0.0019 (4)
C6	0.0126 (4)	0.0127 (4)	0.0140 (4)	−0.0014 (3)	−0.0001 (4)	0.0000 (4)
C7	0.0114 (4)	0.0134 (4)	0.0140 (5)	−0.0009 (3)	−0.0008 (3)	−0.0013 (4)
C8	0.0105 (4)	0.0142 (4)	0.0136 (4)	−0.0001 (3)	0.0007 (3)	0.0006 (4)
C9	0.0102 (4)	0.0130 (4)	0.0149 (4)	−0.0006 (3)	−0.0001 (3)	0.0000 (3)
C10	0.0101 (4)	0.0142 (4)	0.0172 (5)	0.0002 (3)	−0.0010 (4)	−0.0003 (4)
C11	0.0128 (4)	0.0140 (4)	0.0142 (4)	0.0012 (3)	−0.0004 (4)	0.0000 (4)
C12	0.0127 (4)	0.0170 (5)	0.0170 (5)	0.0005 (4)	0.0017 (4)	0.0030 (4)
C13	0.0113 (4)	0.0181 (5)	0.0177 (5)	0.0007 (4)	0.0016 (4)	0.0028 (4)
C14	0.0151 (5)	0.0143 (4)	0.0147 (5)	0.0008 (3)	−0.0012 (4)	−0.0005 (4)
C15	0.0261 (6)	0.0183 (5)	0.0236 (6)	0.0020 (5)	−0.0020 (5)	0.0058 (5)
C16	0.0397 (8)	0.0215 (6)	0.0288 (7)	0.0028 (6)	0.0070 (6)	−0.0002 (5)
C17	0.0101 (4)	0.0204 (5)	0.0165 (5)	0.0025 (4)	−0.0005 (4)	0.0039 (4)
C18	0.0166 (5)	0.0265 (6)	0.0407 (8)	−0.0035 (5)	−0.0102 (5)	0.0018 (6)
C19	0.0214 (6)	0.0409 (8)	0.0248 (7)	0.0142 (6)	0.0012 (5)	−0.0047 (6)
C20	0.0176 (6)	0.0428 (9)	0.0310 (8)	−0.0059 (6)	−0.0073 (5)	0.0231 (7)
C21	0.0339 (7)	0.0139 (5)	0.0220 (6)	0.0002 (5)	0.0013 (5)	0.0024 (4)

### Geometric parameters (Å, °)

**Table:**

O1—C3	1.3649 (15)	C11—C12	1.4102 (16)
O1—C21	1.4277 (17)	C11—C14	1.4833 (16)
O2—C14	1.2174 (15)	C12—C13	1.3860 (16)
O3—C14	1.3432 (16)	C12—H12A	0.9300
O3—C15	1.4509 (16)	C13—H13A	0.9300
N1—C8	1.3908 (15)	C15—C16	1.499 (2)
N1—C7	1.3958 (15)	C15—H15A	0.9700
N1—C17	1.5061 (15)	C15—H15B	0.9700
N2—C7	1.3176 (15)	C16—H16A	0.9600
N2—C9	1.3845 (15)	C16—H16B	0.9600
C1—C6	1.3912 (16)	C16—H16C	0.9600
C1—C2	1.3954 (17)	C17—C19	1.523 (2)
C1—H1A	0.9300	C17—C20	1.5236 (19)
C2—C3	1.3970 (18)	C17—C18	1.5330 (19)
C2—H2A	0.9300	C18—H18A	0.9600
C3—C4	1.3966 (17)	C18—H18B	0.9600
C4—C5	1.3868 (16)	C18—H18C	0.9600
C4—H4A	0.9300	C19—H19A	0.9600
C5—C6	1.4011 (17)	C19—H19B	0.9600
C5—H5A	0.9300	C19—H19C	0.9600
C6—C7	1.4792 (16)	C20—H20A	0.9600
C8—C13	1.4010 (16)	C20—H20B	0.9600
C8—C9	1.4117 (15)	C20—H20C	0.9600
C9—C10	1.3941 (16)	C21—H21A	0.9600
C10—C11	1.3921 (16)	C21—H21B	0.9600
C10—H10A	0.9300	C21—H21C	0.9600
			
C3—O1—C21	117.44 (11)	O2—C14—O3	124.05 (11)
C14—O3—C15	118.20 (11)	O2—C14—C11	124.57 (11)
C8—N1—C7	105.00 (9)	O3—C14—C11	111.38 (10)
C8—N1—C17	124.22 (9)	O3—C15—C16	109.43 (13)
C7—N1—C17	130.60 (10)	O3—C15—H15A	109.8
C7—N2—C9	104.95 (10)	C16—C15—H15A	109.8
C6—C1—C2	121.12 (11)	O3—C15—H15B	109.8
C6—C1—H1A	119.4	C16—C15—H15B	109.8
C2—C1—H1A	119.4	H15A—C15—H15B	108.2
C1—C2—C3	118.93 (11)	C15—C16—H16A	109.5
C1—C2—H2A	120.5	C15—C16—H16B	109.5
C3—C2—H2A	120.5	H16A—C16—H16B	109.5
O1—C3—C4	115.29 (11)	C15—C16—H16C	109.5
O1—C3—C2	124.26 (11)	H16A—C16—H16C	109.5
C4—C3—C2	120.46 (11)	H16B—C16—H16C	109.5
C5—C4—C3	119.80 (11)	N1—C17—C19	108.05 (11)
C5—C4—H4A	120.1	N1—C17—C20	112.78 (10)
C3—C4—H4A	120.1	C19—C17—C20	110.03 (13)
C4—C5—C6	120.42 (11)	N1—C17—C18	109.01 (10)
C4—C5—H5A	119.8	C19—C17—C18	110.85 (13)
C6—C5—H5A	119.8	C20—C17—C18	106.14 (12)
C1—C6—C5	119.08 (11)	C17—C18—H18A	109.5
C1—C6—C7	120.58 (10)	C17—C18—H18B	109.5
C5—C6—C7	120.29 (10)	H18A—C18—H18B	109.5
N2—C7—N1	113.78 (10)	C17—C18—H18C	109.5
N2—C7—C6	120.71 (10)	H18A—C18—H18C	109.5
N1—C7—C6	125.36 (10)	H18B—C18—H18C	109.5
N1—C8—C13	133.18 (10)	C17—C19—H19A	109.5
N1—C8—C9	106.04 (10)	C17—C19—H19B	109.5
C13—C8—C9	120.74 (10)	H19A—C19—H19B	109.5
N2—C9—C10	128.47 (10)	C17—C19—H19C	109.5
N2—C9—C8	110.13 (10)	H19A—C19—H19C	109.5
C10—C9—C8	121.39 (10)	H19B—C19—H19C	109.5
C11—C10—C9	117.71 (10)	C17—C20—H20A	109.5
C11—C10—H10A	121.1	C17—C20—H20B	109.5
C9—C10—H10A	121.1	H20A—C20—H20B	109.5
C10—C11—C12	120.73 (10)	C17—C20—H20C	109.5
C10—C11—C14	118.74 (10)	H20A—C20—H20C	109.5
C12—C11—C14	120.53 (10)	H20B—C20—H20C	109.5
C13—C12—C11	121.94 (11)	O1—C21—H21A	109.5
C13—C12—H12A	119.0	O1—C21—H21B	109.5
C11—C12—H12A	119.0	H21A—C21—H21B	109.5
C12—C13—C8	117.39 (10)	O1—C21—H21C	109.5
C12—C13—H13A	121.3	H21A—C21—H21C	109.5
C8—C13—H13A	121.3	H21B—C21—H21C	109.5
			
C6—C1—C2—C3	−0.48 (19)	C7—N2—C9—C8	−0.18 (13)
C21—O1—C3—C4	176.78 (12)	N1—C8—C9—N2	2.09 (13)
C21—O1—C3—C2	−3.48 (19)	C13—C8—C9—N2	−175.77 (11)
C1—C2—C3—O1	177.04 (12)	N1—C8—C9—C10	−178.71 (11)
C1—C2—C3—C4	−3.22 (18)	C13—C8—C9—C10	3.43 (18)
O1—C3—C4—C5	−176.84 (11)	N2—C9—C10—C11	178.14 (12)
C2—C3—C4—C5	3.40 (18)	C8—C9—C10—C11	−0.90 (18)
C3—C4—C5—C6	0.13 (18)	C9—C10—C11—C12	−1.53 (18)
C2—C1—C6—C5	3.95 (18)	C9—C10—C11—C14	178.49 (11)
C2—C1—C6—C7	−178.63 (11)	C10—C11—C12—C13	1.53 (19)
C4—C5—C6—C1	−3.76 (17)	C14—C11—C12—C13	−178.48 (12)
C4—C5—C6—C7	178.81 (11)	C11—C12—C13—C8	0.94 (19)
C9—N2—C7—N1	−1.88 (14)	N1—C8—C13—C12	179.47 (12)
C9—N2—C7—C6	173.81 (11)	C9—C8—C13—C12	−3.35 (18)
C8—N1—C7—N2	3.19 (14)	C15—O3—C14—O2	−1.2 (2)
C17—N1—C7—N2	−172.05 (11)	C15—O3—C14—C11	179.23 (11)
C8—N1—C7—C6	−172.26 (11)	C10—C11—C14—O2	4.03 (19)
C17—N1—C7—C6	12.49 (19)	C12—C11—C14—O2	−175.95 (13)
C1—C6—C7—N2	−110.78 (14)	C10—C11—C14—O3	−176.36 (11)
C5—C6—C7—N2	66.61 (15)	C12—C11—C14—O3	3.65 (16)
C1—C6—C7—N1	64.39 (16)	C14—O3—C15—C16	−97.76 (15)
C5—C6—C7—N1	−118.22 (13)	C8—N1—C17—C19	76.40 (15)
C7—N1—C8—C13	174.46 (13)	C7—N1—C17—C19	−109.15 (15)
C17—N1—C8—C13	−9.9 (2)	C8—N1—C17—C20	−161.76 (13)
C7—N1—C8—C9	−3.02 (12)	C7—N1—C17—C20	12.69 (19)
C17—N1—C8—C9	172.61 (11)	C8—N1—C17—C18	−44.15 (16)
C7—N2—C9—C10	−179.31 (12)	C7—N1—C17—C18	130.30 (14)

### Hydrogen-bond geometry (Å, °)

**Table:**

Cg1, Cg2 and Cg3 are the centroids of the rings N1,N2,C7–C9 and C1–C6 and C8–C13, respectively.

**Table:**

*D*—H···*A*	*D*—H	H···*A*	*D*···*A*	*D*—H···*A*
C5—H5A···O2^i^	0.93	2.57	3.3822 (16)	146
C13—H13A···O2^ii^	0.93	2.52	3.4116 (16)	160
C19—H19C···N2^iii^	0.96	2.57	3.4976 (19)	164
C2—H2A···Cg3^iv^	0.93	2.75	3.5594 (14)	146
C18—H18C···Cg2^v^	0.96	2.74	3.6878 (16)	168
C21—H21B···Cg1^iv^	0.96	2.78	3.4703 (16)	130

Symmetry codes: (i) −*x*+1/2, *y*+1/2, *z*−1/2; (ii) *x*−1/2, −*y*+1/2, *z*; (iii) *x*−1/2, −*y*+3/2, *z*; (iv) *x*, *y*+1, *z*; (v) −*x*−1/2, *y*+3/2, *z*+1/2.
